# Reprogramming the host: Mycobacterium tuberculosis as a silent architect of the immuno-tumoral

**DOI:** 10.3389/fcimb.2025.1697874

**Published:** 2025-11-13

**Authors:** Rodolfo L. Chávez-Domínguez, Martha Torres, Atziri A. Acevedo-Domínguez, Jesús A. Ibarra-Inocente, Claudia Carranza

**Affiliations:** Laboratorio de Inmunobiología de la Tuberculosis, Instituto Nacional de Enfermedades, Respiratorias (INER) Ismael Cosío Villegas, Mexico City, Mexico

**Keywords:** *Mycobacterium tuberculosis*, lung cancer, chronic inflammation, carcinogenesis, immune-tumoral

## Abstract

Pulmonary tuberculosis, caused by *Mycobacterium tuberculosis* (Mtb), remains one of the leading causes of infectious disease-related mortality worldwide. In parallel, lung cancer represents the most lethal neoplasm, with high mortality rates globally. Emerging studies suggest that chronic Mtb infection may contribute to the development of lung cancer, particularly adenocarcinoma. Several biological mechanisms support this hypothesis. Chronic inflammation from tuberculosis creates a microenvironment enriched in proinflammatory cytokines, reactive oxygen species (ROS), and growth factors that favor cell proliferation, genomic instability, angiogenesis, and immune evasion, which are considered classic hallmarks of cancer. Additionally, both protein and non-protein virulence factors of Mtb have been shown to interfere with critical cellular signaling pathways related to tumor cell survival and invasion. Clinically, multiple observational studies and meta-analyses report an increased incidence of lung cancer among individuals with a history of tuberculosis, especially when both conditions coexist in the same pulmonary regions. Specific mutations, including EGFR, have been identified in patients with prior tuberculosis, influencing both prognosis and therapeutic response. Nevertheless, key questions remain regarding the causal nature of this association, the role of Mtb strains, and the molecular factors such as epigenetic modifications or the lung microbiome. This review proposes that infection with Mtb could function as a carcinogenic agent. Further *in vitro* experiments, cellular models, and clinical investigations are urgently needed to support potential reclassification of this pathogen by international agencies such as the IARC.

## Graphical Abstract

Association between Mycobacterium tuberculosis infection, chronic inflammation, and lung cancer development. Chronic infection with Mycobacterium tuberculosis induces granuloma formation and fibrotic lesions in the lung (left). Persistent inflammation and the release of mycobacterial virulence factors, together with soluble mediators from immune cells, contribute to the dysregulation of key signaling pathways involved in the hallmarks of cancer (middle). Clinically, lung adenocarcinoma and other malignancies often arise in the vicinity of granulomas, fibrotic scars, and areas of chronic tissue damage, and are associated with driver gene mutations and immune checkpoint expression (right).

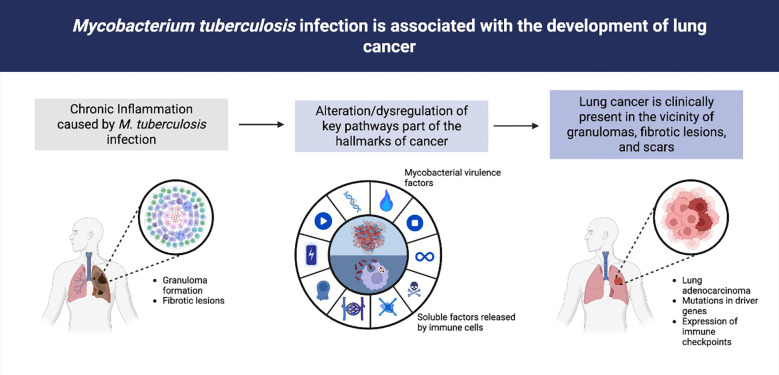

## Introduction

Pulmonary tuberculosis (TB) is caused by the Mycobacterium tuberculosis complex. A related group of pathogens, nontuberculous mycobacteria (NTM), can produce a similar clinical syndrome and must be distinguished due to differing diagnostic and treatment implications. Tuberculosis remains one of the leading causes of death associated with infectious agents. The World Health Organization’s 2024 Global Tuberculosis Report estimates that approximately 1.25 million people died from tuberculosis in 2023 (Global tuberculosis report 2024, 2024). Furthermore, it is estimated that around one-third of the global population has been infected with Mtb yet without presenting clinical manifestations, a condition referred to as latent tuberculosis infection (Cohen et al., 2019). However, certain factors such as metabolic disorders and immunosuppressive conditions may lead to reactivation of the infection in latently infected individuals or increase susceptibility to primary infection.

Regarding lung cancer (LC), this disease ranks as the leading cause of cancer-related mortality worldwide in both men and women (Bray et al., 2024). In Mexico and Latin America, it is estimated that approximately 100,000 individuals die annually from LC, establishing this pathology as a major global public health concern (Piñeros et al., 2022). Most cases are diagnosed at advanced stages. Non-small cell lung cancer (NSCLC) accounts for approximately 85% of all lung cancers, with pulmonary adenocarcinoma being the most frequently observed histological subtype (representing approximately 40% of all lung cancers), followed by squamous cell carcinoma (25-30%) and large cell carcinoma (10-15%). Small cell lung cancer (SCLC) comprises the remaining 10-15% of cases (Budisan et al., 2021). Major risk factors include tobacco smoking, exposure to wood smoke, and infection with certain pathogenic microorganisms such as *Chlamydophila pneumoniae*, *Cryptococcus* spp., *Helicobacter pylori*, and *Mycobacterium tuberculosis* (Budisan et al., 2021).

In the past decade, tumor-associated inflammatory response has been increasingly recognized as a paradoxical effect on tumorigenesis and progression (Hanahan and Weinberg, 2011). Pathogen-induced inflammation has been identified as a condition associated with the onset of various malignancies. The International Agency for Research on Cancer (IARC) has classified infections by 19 pathogenic agents, including 7 viruses, 3 nematodes, and one bacterium, as Group I carcinogens. Notably, Mtb infection is not currently included within this classification (List of classifications).

Nonetheless, the relationship between tuberculosis and LC has been reported in numerous studies, demonstrating an epidemiological association between these two diseases (Wong et al., 2020; Zhang et al., 2025).

Pulmonary fibrotic lesions caused by exacerbated inflammation during tuberculosis infection have been suggested as predisposing factors for LC development (Bhatt et al., 2012). Additionally, the diagnostic process for these diseases is often complicated, as both share clinical symptoms such as fever, productive cough, hemoptysis, weight loss, and anorexia (Bhatt et al., 2012).

Most studies, particularly those conducted in Asian populations, report a 3 to 8 fold increase in the risk of developing LC, especially adenocarcinoma, among patients with chronic forms of tuberculosis (Abdeahad et al., 2022). Although LC risk has been assessed broadly in patients with past or current tuberculosis, there has been no separate analysis of latent versus active infection. This represents a critical knowledge gap, as latency and active disease likely confer distinct inflammatory and immunological milieus that may differentially influence carcinogenesis. Future prospective studies should stratify TB cohorts by latent versus active status to compare subsequent LC incidence and clarify whether risk differs by infection phase.

LC patients infected with Mtb often exhibit poorer prognoses and reduced overall survival compared to uninfected individuals (Lee et al., 2022).

However, current evidence regarding tuberculosis and LC association remains inconclusive, as no comprehensive framework exists to integrate findings related to the precise molecular mechanisms by which Mtb may contribute to tumorigenesis. Despite clinical data supporting pulmonary tuberculosis and cancer risk, Mtb has yet to be classified by IARC as a carcinogenic agent, an omission that holds important implications for LC prevention and early diagnosis.

This review aims to explore the association between Mtb infection and the risk of developing LC. It will examine the molecular mechanisms underlying this relationship, integrating them with the canonical hallmarks of cancer. Furthermore, we will assess existing clinical evidence supporting the tuberculosis-cancer link, considering histopathological, molecular, and immunological aspects of tumor progression. Finally, unresolved questions and research gaps within this complex interplay will be discussed.

## Pathogenesis of tuberculosis and inflammation

Recent data following the SARS-CoV-2 pandemic show a resurgence in global tuberculosis incidence and mortality (Scriba et al., 2024). Tuberculosis is now the leading cause of death by an infectious agent (Global tuberculosis report 2024, 2024).

Mtb is a highly adaptable bacterium that has co-evolved with humans, acquiring mechanisms to evade and modulate host immune responses, thereby enabling its long-term persistence within the host (Abbasnia et al., 2024). Tuberculosis is not merely the consequence of infection with Mtb but rather the result of complex interactions between the pathogen and the host immune system. These interactions may result in a wide spectrum of clinical manifestations, ranging from asymptomatic infection to active disease, involving pulmonary and extrapulmonary sites (Scriba et al., 2024).

Mtb infection is initiated when bacilli are inhaled and subsequently phagocytosed by alveolar macrophages, the first line of immune defense. During phagocytosis, various receptors, including complement receptors, mannose receptors, and Fc receptors, are involved (Mohammadnabi et al., 2024). Once internalized, these macrophages migrate into the pulmonary interstitium, recruiting additional macrophages and immune cell subsets to control the infection (Cronan, 2022). However, Mtb evades elimination by interfering with phagosomal maturation and acidification and with phagolysosomal fusion (Queval et al., 2017; Xander et al., 2024). Within the phagolysosome, Mtb survives through the activation of antioxidant enzymes that neutralize reactive oxygen species (ROS) and reactive nitrogen species (RNS), promoting replication and further infection of host macrophages (Ehrt and Schnappinger, 2009; Pal et al., 2024).

At this stage, numerous immune cells are recruited to the infection site, including dendritic cells, neutrophils, epithelioid macrophages, NK cells, CD4+ and CD8+ T cells, and fibroblasts. This recruitment leads to granuloma formation (Hunter et al., 2006; Queval et al., 2017). Granulomas are not exclusively the result of host immune attempts to contain and prevent dissemination; Mtb also produces factors that promote granuloma formation (Cronan, 2022; Carabalí-Isajar et al., 2023). Notably, studies have shown that infected macrophages release the mycobacterial ESAT-6 protein, inducing the expression of matrix metalloproteinase 9 (MMP9) in epithelial cells (Volkman et al., 2010). MMP9 facilitates the recruitment of additional macrophages to the infection site, contributing to granuloma expansion (Volkman et al., 2010). Moreover, Mtb-derived lipid components, such as 6,6-trehalose dimycolate, are implicated in the structural assembly of granulomas (Hunter et al., 2006).

Within these structures, bacilli replicate while being targeted by immune cells, establishing a dynamic equilibrium that limits dissemination. This explains latent infection in approximately 95% of exposed individuals (Dutta and Karakousis, 2014). However, immune compromise can disrupt this equilibrium, leading to infection reactivation. Conditions include malnutrition, immunodeficiencies, diabetes, smoking, and chronic pulmonary diseases (Ai et al., 2016; Girmay et al., 2024), leading to reactivation of the infection. Mtb infection induces inflammatory cytokines, chemokines, growth factors, and other immune-derived molecules. In this inflammatory microenvironment, cytokines such as TNF-α, IFN-γ, and IL-6 activate anti-apoptotic gene expression through the nuclear factor kappa B (NF-κB) signaling pathway in pulmonary epithelial cells, which may promote carcinogenesis.

## Inflammation and cancer

Inflammation is the biological and nonspecific response caused by infection or biological substance, encompassing the control of these factors and the subsequent regeneration of injured tissue (Hibino et al., 2021). However, continuous activation of the inflammatory process and the failure to resolve it are associated with pathological conditions, including cancer (Michels et al., 2021). The inflammation-cancer link has been documented since the early 19th century. Jean Nicolas Marjolin and Rudolf Virchow reported inflammation induced tissue damage in neoplastic lesions and immune cell infiltration into tumors (Liu et al., 2023). A century later, defined tumors as “wounds that do not heal,” emphasizing the similarity between tumor development and the tissue damage and regeneration cycle inherent to inflammation (Dvorak, 1986). Based on extensive research, inflammation is currently regarded as a phenomenon that actively promotes tumor development (Hanahan, 2022).

Chronic inflammation promotes carcinogenesis through multiple interrelated mechanisms. Persistent cytokine and chemokine signaling (TNF-α, IL-6) activates transcription factors such as NF-κB and STAT3, which upregulate anti-apoptotic genes and cell cycle regulators, fostering survival and proliferation of mutated cells. Simultaneously, sustained generation of reactive oxygen and nitrogen species by inflammatory cells induces DNA damage and genomic instability, increasing mutation rates. Inflammation also drives angiogenesis via upregulation of VEGF and matrix metalloproteinases, facilitating nutrient delivery and tumor invasion. Finally, inflammatory mediators remodel the extracellular matrix and induce EMT, enhancing metastatic potential. Together, these processes illustrate how a chronic inflammatory microenvironment can initiate and promote malignant transformation.

Depending on the cause triggering the inflammatory process, inflammation is classified as either sterile or non-sterile. WHO reports indicate that approximately 15% of all cancers are attributable to infectious agents, of which 9.9% are viruses (Plummer et al., 2016). In Latin America, it is estimated that roughly 150,000 cancer cases are caused by infections (Espina et al., 2023). Among the principal infectious agents recognized as contributors to various malignancies are *Helicobacter pylori*, *Fusobacterium nucleatum*, Schistosoma haematobium, Streptococcus bovis, human immunodeficiency virus (HIV), human papillomavirus (HPV), hepatitis B and C viruses, among others (Liu et al., 2023). These pathogens have been proposed to act as direct or indirect carcinogens (Vandeven and Nghiem, 2014).

Direct carcinogenic pathogens can integrate into the host cell genome and promote the expression of oncogenes. In contrast, infections caused by indirect carcinogenic pathogens do not directly activate oncogenes. These agents induce a chronic inflammatory state in which pathogen-associated molecular patterns (PAMPs) are recognized by pattern recognition receptors (PRRs), such as Toll-like receptors (TLRs) expressed by various immune cells (Li and Wu, 2021). Activation of these cells leads to the release of proinflammatory cytokines and chemokines, including TNF-α, IL-6, IL-1β, and IL-8 (Finlay and McFadden, 2006).

In the case of *Helicobacter pylori*, infection in the gastric epithelium has been associated with elevated production and release of reactive oxygen species (ROS), which damage host DNA. Additionally, ROS trigger pathways that stabilize signal transducer and activator of transcription 3 (STAT3), promoting the expression of proinflammatory cytokines such as IL-6 and IL-11 (Prasad et al., 2022). Similarly, pulmonary infections with Chlamydophila pneumoniae have been linked to lung carcinogenesis via increased release of IL-6, TNF-α, and IL-1β (Budisan et al., 2021).

Furthermore, infectious agents can synthesize molecular components intrinsic to their replication cycles that simultaneously activate oncogenes or inactivate tumor suppressor genes (Ameya and Birri, 2023). E6 and E7 proteins of HPV promote resistance to cell death and inhibit antiproliferative signals in epithelial cells by targeting the tumor suppressor protein TP53 for degradation (Ansari et al., 2012). Bacterial plasmid mucAB and its Escherichia coli genomic homolog umuDC increase neoplastic lesions in murine models. These plasmids sequester microRNA-145, a tumor suppressor that regulates oncogenes such as Nedd9 and Aurkb, thereby promoting the transformation of infected cells toward a tumoral phenotype (Tanooka et al., 2020). In addition, virulence factors such as cytotoxin-associated gene A (CagA), vacuolating cytotoxin A (VacA), and outer inflammatory protein A (OipA) produced by Helicobacter pylori have been identified as initiators of tumorigenic processes (Elagan et al., 2021).

These findings raise the question of whether molecular products derived from Mycobacterium tuberculosis (Mtb) or its virulence factors may likewise contribute to epithelial cell transformation, thereby explaining the increased incidence of cancer in individuals with chronic Mtb infections.

## Molecular relationship between tuberculosis and lung cancer

The comorbidity of TB and cancer has been documented in numerous prospective, retrospective, and meta-analytical studies. The risk is higher for men and much higher for the elderly (Yu et al., 2011). Recent work employing cellular and animal models has offered an initial glimpse into the possible nature of this association. Mtb protein tyrosine phosphatase (PtpA) is a key effector protein that enters the host cell nucleus and upregulates MK167 host gene expression. This activation leads to uncontrolled proliferation, evasion of cell death, and induction of epithelial-to-mesenchymal transition (EMT) in Mtb infected human lung adenoma A549 cell (Chai et al., 2020).

Furthermore, Mtb possesses diverse virulence factors that allow it to infect the host, induce pulmonary damage, evade immune responses, and facilitate transmission to new hosts (Virulence factors and pathogenicity of mycobacterium, 2018). These virulence factors may also contribute to cellular transformation and tumorigenesis. They have been categorized into protein and non-protein types (Ramon-Luing et al., 2023).

The non-protein virulence factors can regulate diverse modes of cell death. Depending on biological context, they may either promote or suppress apoptosis (Ramon-Luing et al., 2023). Man-LAM has been shown to upregulate anti-apoptotic Bcl-2 family proteins and downregulate Bax in murine macrophages, thereby inhibiting apoptosis (Wojtas et al., 2011).

Mtb virulence factors activate oncogenic processes such as proliferation, resistance to apoptosis, and invasion programming and upregulate transcription factors that induce epithelial to mesenchymal transition (EMT), a signal of carcinogenesis and metastatic progression (Malik et al., 2022). For instance, the nuoG protein interferes with TNF-α induced pro-apoptotic signaling by neutralizing ROS and RNS (Miller et al., 2010). Serine/threonine kinases produced by Mtb also suppress apoptosis; notably, the PknE protein inhibits intrinsic apoptosis triggered by RNS by reducing Bax and Bid expression (Kumar and Narayanan, 2012). Collectively, these findings suggest that Mtb virulence factors may enhance the survival of early tumor cells by suppressing apoptotic pathways.

Although further studies in non-transformed epithelial cells are needed, this phenomenon suggests that Mtb derived molecules may directly contribute to cancer development. While these studies imply that Mtb virulence factors support transformation to a tumoral phenotype, the impact of distinct virulent strains on cancer progression remains unknown. Recently, Hadifar et al. showed that infection of A549 cells with two genetically distinct Mtb strains from Iran (L3-CAS1 and L4.5) differentially induced genes associated with proliferation, immune evasion, and inflammation (Hadifar et al., 2021).

Although these experiments were conducted in a tumor-derived cell line, they open the possibility of examining strain-specific effects in healthy epithelial cells.

In addition, Mtb behaves as an indirect carcinogenic pathogen due to the influence of inflammatory cytokines, chemokines, growth factors, and other immune-derived molecules produced during infection that contribute to tumor development. In this inflammatory microenvironment, cytokines such as TNF-α, IFN-γ, and IL-6 activate anti-apoptotic gene expression through the nuclear factor kappa B (NF-κB) signaling pathway in pulmonary epithelial cells, which may promote carcinogenesis (Scriba et al., 2024). Moreover, Mtb induces substantial ROS and RNS production by activated phagocytes. Although these molecules are essential for bacterial clearance, their excessive production leads to tissue damage and oxidative stress conditions that can drive tumorigenesis through DNA damage (Abbasnia et al., 2024).

Specifically, macrophages infected with Mtb release pro-inflammatory cytokines, which, in the context of cancer, contribute to enhanced cellular proliferation, evasion of cell death, and activation of signaling pathways that promote invasion and metastasis (Holla et al., 2014; Zhang et al., 2015; Gupta et al., 2016). Interestingly, Mtb infected macrophages produce epidermal growth factor epiregulin, a potent ligand of the epidermal growth factor family involved in squamous metaplasia and tumorigenesis (Nalbandian et al., 2009). Additionally, studies using Mtb susceptible mouse models Mtb demonstrate that, during chronic infection, macrophages cause DNA damage in pulmonary epithelial cells through excessive generation of reactive oxygen species (ROS) and reactive nitrogen species (RNS). This oxidative stress fosters genomic instability and the accumulation of mutations in proto-oncogenes and tumor suppressor genes (Da Silva et al., 2015; Ghosh et al., 2019).

Another critical aspect is the induction of angiogenesis, new blood vessel formation from existing vasculature triggered by Mtb infection. While angiogenesis facilitates immune cell recruitment to the granuloma to contain the infection, it is also considered a mechanism that fosters bacterial dissemination to distant sites (Maison, 2022). *In vitro* studies have shown that Mtb-infected macrophages upregulate genes associated with angiogenesis, including those encoding vascular endothelial growth factor A (VEGF-A), angiogenin, and matrix metalloproteinases 1, 3, and 10 (Polena et al., 2016).

During infection, VEGF-A expression in macrophages is regulated by activation of the transcription factor NFAT (Brewer et al., 2022). Notably, expression levels of angiogenic mediators increase with the virulence of the Mtb strain. In murine models, blood vessel formation occurs near the site of infection, suggesting that Mtb driven angiogenesis not only promotes tumor cell growth and survival but may also facilitate their dissemination.

Mtb infection also influences cellular and non-cellular components of the tumor microenvironment. In advanced infection stages, macrophages polarize toward an M2 phenotype, characterized by secretion of immunomodulatory cytokines such as transforming growth factor-beta (TGF-β), interleukin-10 (IL-10), and Th2-type cytokines. In cancer, these molecules are involved in cell proliferation and epithelial-to-mesenchymal transition (EMT), processes that underpin tumor invasion and metastasis (Xiong et al., 2021). *In vitro* studies have reported that Mtb infected macrophages secrete IL-1β, TNF-α, and IL-6, which induce EMT in pulmonary adenocarcinoma cells, thereby enhancing their invasive and metastatic potential (Gupta et al., 2016).

Metabolic reprogramming is one of the hallmarks of cancer, wherein uncontrolled proliferation leads tumor cells to preferentially utilize glucose via glycolysis with the formation and secretion of lactate even under normoxic conditions, a phenomenon termed ‘aerobic glycolysis’ or the Warburg effect (List of classifications).

Animal models have demonstrated that pulmonary Mtb infection induces similar glycolytic metabolic shifts, with increased expression of glycolytic enzymes and glucose transporters (Cao et al., 2019). In clinical samples from patients with active TB, genes encoding glucose transporters GLUT1 and GLUT3, hexokinases HK1 and HK3, and monocarboxylate transporters are upregulated (Huang et al., 2025). This metabolic reprogramming occurs in multiple cellular compartments, including immune cells (particularly activated T lymphocytes and macrophages), infected epithelial cells, and within the granuloma structure itself (Cao et al., 2019; Huang et al., 2025).

However, it is important to note that the observed glycolytic upregulation in TB-infected tissue primarily reflects the metabolic demands of activated immune cells responding to infection, rather than directly indicating tumor-promoting metabolic changes (Cao et al., 2019; Huang et al., 2025). Activated T cells and macrophages physiologically upregulate aerobic glycolysis to support rapid proliferation, cytokine production, and antimicrobial functions. While this Mtb-induced metabolic landscape shares features with the tumor microenvironment, including elevated glucose consumption, lactate production, and hypoxic conditions, whether this metabolic reprogramming causally contributes to tumor development or merely represents an immune response remains uncertain. The temporal relationship between TB-associated metabolic changes and subsequent malignant transformation, and whether persistent metabolic alterations following TB resolution create lasting pro-tumorigenic conditions, require further investigation through longitudinal studies in appropriate model systems. Therefore, while Mtb infection clearly induces a Warburg-like metabolic state, concluding that this directly promotes tumor microenvironment formation would be premature given current evidence.

Mtb Additionally, it has been shown that Mtb infection promotes immune evasion by fostering the development of an immunosuppressive environment within the tumor (Zhou et al., 2017). The tumor microenvironment (TME) and tuberculous granulomas share several common features, including macrophage polarization programs toward the M2 phenotype and the presence of exhausted T cell phenotypes (Scriba et al., 2024). Both conditions contribute to a locally immunosuppressive milieu that supports bacterial persistence in TB and tumor progression in cancer.

Inflammatory signals within this microenvironment promote immunosuppression through mechanisms such as the differentiation of T lymphocytes into regulatory T cells (Tregs), the expansion of myeloid-derived suppressor cells (MDSCs), and other immunoregulatory components. These signals also enhance the recruitment, proliferation, and functional specialization of various pro-tumorigenic helper cell subsets within the TME (Magcwebeba et al., 2019). For instance, studies have reported that the co-occurrence of LC and Mtb infection increases the proportion of Treg cells in *in vitro* models. Mtb infected macrophages upregulate immunomodulatory molecules such as PD-L1, which interact with naïve CD4^+^ T cells to induce their differentiation into Tregs cells that facilitate and promote tumor development (Zhou et al., 2017).

Interestingly, the expression of PD-L1, PD-1, and PD-L2 is not restricted to infected macrophages; peripheral blood T cells from Mtb infected patients also exhibit increased expression of these immune checkpoint molecules (Cao et al., 2019).

Multiple investigations have identified overlapping molecular mechanisms activated during Mtb infection that converge with critical pathways in carcinogenesis. Sustained inflammation, apoptotic dysregulation, metabolic reprogramming, and immune evasion represent shared nodes between both pathophysiological contexts. **Table 1** summarizes the cancer hallmarks linked to components derived from the inflammatory and microbial response, highlighting the implicated molecules and signaling pathways that may contribute to malignant transformation.

**Table 1:** Cancer hallmarks influenced by immunological and metabolic factors derived from *Mycobacterium tuberculosis* infection.

Cancer hallmark	Key associated molecules	Linked mechanism
Chronic inflammation	TNF-α, IL-1β, IL-6, IL-8, IL-11, NF-κB, STAT3	Release of proinflammatory cytokines that promote proliferation and angiogenesis
Apoptosis evasion	↑Bcl-2, ↓Bax, ↓Bid, nuoG, PknE, Man-LAM	Inhibition of programmed cell death, favoring survival of abnormal cells
Sustained proliferation	Mutated EGFR, Epiregulin, ptpA, STAT3	Activation of proliferative pathways in infected epithelial cells
Angiogenesis	VEGF-A, Angiogenin, MMPs, NFAT	Induction of blood vessel formation that nourishes the tumor and facilitates dissemination
Invasion and metastasis	MMP-9, MMP-10, IL-6, EMT (induced by IL-1β, TNF-α)	Tissue remodeling and phenotypic switching that enable cellular migration
Metabolic reprogramming	GLUT1, GLUT3, HK1, HK3, MCTs	Induction of the Warburg effect in the infectious-tumoral microenvironment
Immune evasion	PD-1, PD-L1, CTLA-4, IL-10, TGF-β, ↑Treg cells, M2 phenotype	Suppression of antitumor immune responses, promoting a tolerogenic microenvironment
Genomic instability	ROS, RNS, miR-145 sequestration, Nedd9, Aurkb	DNA damage and epigenetic alterations contributing to malignant transformation

## Clinical and molecular evidence linking *Mycobacterium tuberculosis* infection to lung cancer

Although most studies have focused on advanced stages of tumor development, current findings support the hypothesis that Mtb acts as a potential oncogenic pathogen. However, its impact on other cancer related characteristics such as epigenetic regulation, phenotypic plasticity, replicative immortality, and microbiome alterations remains underexplored.

A systematic literature search was conducted in the PubMed, Web of Science, and Google Scholar databases, covering the period from January 2010 to July 2025. The search strategy included the following MeSH terms and keywords to retrieve information: “carcinogenesis AND tuberculosis,” “lung cancer AND pulmonary tuberculosis,” and “lung cancer AND active tuberculosis.” Reviews, meta-analyses, original research, and case reports were included.

The co-occurrence of TB and LC has been documented in various clinical studies and case reports, where both diseases may present simultaneously, complicating diagnosis and clinical management (Yu et al., 2021; Huang et al., 2025). Alternatively, they may arise independently, with one increasing the risk of the other. In this context, clinical research has focused on evaluating the risk of LC development in patients with active, chronic, and aggressive TB.

One of the earliest clinical studies conducted in an Asian population demonstrated that TB increases the risk of developing LC by approximately 2.5 fold. Remarkably, this prospective, longitudinal investigation revealed that LC frequently emerged in the same pulmonary region previously affected by TB lesions (Zheng et al., 1987). Follow-up studies, using varied experimental designs and in different populations, have validated these observations. A recent meta-analysis reported that TB significantly raises cancer risk, with the most pronounced effect in LC (Zhang et al., 2008). This elevated risk occurs within five years of TB diagnosis, suggesting that chronic inflammation and infection-related factors contribute to tumor initiation (Luczynski et al., 2022). In addition to M. tuberculosis, NTM species such as the Mycobacterium avium complex can coexist with LC. For example, Mishra et al (Zhang et al., 2008). described a case of pulmonary MAC infection concurrent with adenocarcinoma of the lung. Other studies report NTM isolates in LC patients, underscoring the clinical importance of differentiating TB from NTM-associated disease in prognosis and therapy.

**Table 2** summarizes patient-based clinical studies examining the tuberculosis-LC association. Studies were included if they reported specific numbers of human patients and examined TB-LC relationships through clinical, epidemiological, prognostic, or biomarker analyses. Eligible study designs included retrospective and prospective cohorts, case-control studies, population-based cohorts, cross-sectional studies, systematic reviews with meta-analyses, case series (≥2 patients), case reports with substantial clinical data, and transcriptomic/biomarker studies utilizing patient-derived samples. Studies were excluded if they lacked patient data (pure *in vitro* cell culture, animal models, mechanistic-only investigations), were non-peer reviewed (conference abstracts, preliminary reports), or examined unrelated conditions. The collection encompasses 5 systematic reviews/meta-analyses pooling data from over 5 million participants, 12 retrospective/prospective cohort studies (20-3,567 patients), 5 cross-sectional/case-control studies examining immunological profiles and genetic associations, 4 case reports/series documenting rare presentations, and 3 specialized studies utilizing patient specimens for transcriptomic or biomarker analyses.

**Table 2:** Summary of clinical studies examining the tuberculosis-lung cancer.

Reference	Study type	Number of patients	Key findings
Cohen et al., 2019	Systematic review & meta-analysis	2,609,137 participants (34 studies)	Pooled LTBI prevalence: 24.8% globally; highest in Western Pacific (35.4%) and Southeast Asia (30.6%); important baseline for LC risk
Wong et al., 2020	Mendelian randomization (GWAS data)	6,877 never-smoking Asian women with LUAD (GWAS data)	Mendelian randomization identified genetic variants (MFN2 rs4240897, HLA-DQA1) associating TB with LUAD risk (OR 1.31); shared pathways
Zhang et al., 2025	Population-based matching study	472 patients (236 TB-LC, 236 controls)	TB-LC patients: older (median 68 vs 64 years), more male (92.8% vs 67.4%), more smokers; 60.6% upper lobe lesions; imaging overlaps complicate diagnosis
Bhatt et al., 2012	Retrospective case series	8 PTB cases	TB mimics LC clinically/radiologically; 8 cases initially diagnosed as LC; importance of differential diagnosis emphasized
Abdeahad et al., 2022	Systematic review & meta-analysis	9 studies, varying cohorts	Prior PTB increases LC risk: pooled RR 1.79 (95% CI 1.21-2.63); risk persists long-term; meta-analysis of 9 studies
Lee et al., 2022	Retrospective cohort	62 concurrent LC-PTB patients	Concurrent TB-LC: older age, smoking, COPD risk factors; diagnosis interval affects outcomes; 62 patients analyzed
Girmay et al., 2024	Comparative cross-sectional	210 participants (72 LTBI, 68 HIV+LTBI, 70 controls)	HIV+LTBI patients show impaired Th1 response (reduced IFN-γ, TNF-α) and pro-inflammatory cytokines vs LTBI-only; immunosuppression increases risk
Michels et al., 2021	Systematic review & meta-analysis	1,856,363 participants (26 studies)	Chronic inflammation increases cancer incidence: HR 1.42 (95% CI 1.28-1.58); meta-analysis of 26 epidemiological studies
Plummer et al., 2016	Synthetic analysis (burden estimation)	Global cancer burden data (2012)	2.2 million cancer cases (16%) attributable to infections in 2012; establishes infection-cancer burden; TB not specifically quantified
Yu et al., 2011	Population-based cohort	3,567 PTB patients (117 developed LC)	PTB increases LC risk: adjusted HR 3.3 (95% CI 2.2-5.0); risk highest in first 5 years (HR 4.9); population cohort of 3,567 PTB patients
Chai et al., 2020	Transcriptomic analysis with patient samples	368 lung tissue samples (PTB, LUAD, sarcoidosis patients)	Gene expression profiling identified shared pathways (IL-17, TNF, NF-κB, immune response) between PTB, LUAD, and sarcoidosis; 6 AA metabolism genes discriminate diseases
Da Silva et al., 2015	Cross-sectional	114 participants (32 TB, 32 LC, 50 COPD)	DNA damage (comet assay) and micronuclei significantly higher in TB and LC vs controls; genotoxic mechanisms linking TB to carcinogenesis
Subbian et al., 2015	Pilot study (granuloma analysis)	53 PTB patients (granuloma specimens)	Granuloma-specific immune profiles: central caseous regions show distinct cytokine patterns vs peripheral zones; pilot study in 53 PTB patients
Luo et al., 2012	Retrospective cohort	143 LUAD patients	Old PTB lesions associated with EGFR mutations (50% vs 34.7%, p=0.085); EGFR-mutant patients with PTB had worse PFS (11.7 vs 21.2 months)
Zhang et al., 2020.	Cross-sectional	60 TB patients	BTLA+ dendritic cells in TB patients show reduced IL-12/IFN-α, increased IL-4/TGF-β production, favoring Th2/Treg polarization; 60 TB patients
Saharia et al., 2016	Prospective cohort	25 TB patients (longitudinal)	TB therapy modifies CD4+ T-cell profiles: decreased TNF-α, increased IL-10; longitudinal study in 25 TB patients over treatment course
Huang et al., 2025	Case report with literature review	1 sarcomatoid carcinoma-TB patient	Sarcomatoid carcinoma coexisting with TB in single lesion; rare presentation requiring careful pathological evaluation; case report with review
Yu et al., 2021	Case report	1 SCC-PTB patient	SCC and PTB coexisting within single lesion; diagnostic challenge; successful treatment with sequential TB therapy then surgery; case report
Zheng et al., 1987	Case-control study	213 LC patients	Case-control study (213 LC cases): prior TB significantly associated with LC (OR 1.59, p<0.05); dose-response with TB severity; Shanghai cohort
Luczynski et al., 2022	Systematic review & meta-analysis	1,044,856 patients (24 studies)	Pooled SIR 3.20 (95% CI 2.21-4.63) for LC after TB; highest first year (SIR 4.70), persists >5 years; meta-analysis of 24 studies
Wu et al., 2011	Population-based cohort	716 PTB patients (40 developed LC)	PTB increases LC risk: HR 1.97 (95% CI 1.51-2.58); 40/716 PTB patients developed LC; population-based cohort with mean 6.5-year follow-up
Cicėnas et al., 2007	Retrospective case series	91 TB patients (22 with LC)	Among 91 TB patients, 22 (24.2%) had LC; concurrent presentation common; diagnostic challenges due to overlapping features; retrospective series
Hwang et al., 2019	Retrospective cohort	300 LUAD patients	PTB history associated with higher EGFR mutation rate (46.3% vs 39.9%) and worse OS in EGFR-mutant patients (31.7 vs 42.4 months); 300 LUAD patients
Zhang et al., 2008	Patient cohort study	168 NSCLC patients	Intratumoral epiregulin (marker of advanced disease) higher in EGFR-mutant NSCLC; associated with invasive properties; 168 patients analyzed
Shen et al., 2016a	Cross-sectional	52 active TB patients	PD-1/PD-L pathway activation in active TB inhibits M.tb-specific CD4+ T-cell functions and macrophage phagocytosis; 52 active TB patients vs controls
Shen et al., 2016b	Prospective cohort	20 active TB patients (longitudinal)	PD-1 and PD-L1 expressions elevated in active TB, decline during successful treatment; dynamic monitoring in 20 patients longitudinally
Qiu et al., 2012	Cross-sectional	30 TB patients	Tim-3+ CD4+ and CD8+ T cells in TB exhibit effector memory phenotype and stronger anti-TB functions; 30 TB patients characterized
Chen et al., 2024	Retrospective cohort	1,175 cancer patients receiving ICIs	ICI-treated cancer patients have 5.9-fold increased TB reactivation risk; 13/1175 (1.11%) developed TB; retrospective cohort in Taiwan
Zaemes et al., 2020	Systematic review	92 patients (39 TB cases among ICI users)	TB incidence among ICI users: 0.43% vs general population 0.13%; systematic review of 39 TB cases among 92 reported infections

The increased oncogenic risk has been attributed to chronic inflammation and fibrosis caused by TB in lung tissues. Specifically, fibrotic remodeling creates a microenvironment conducive to the emergence of dysplastic and neoplastic lesions. Hence, TB not only leaves anatomical sequelae but also establishes biological conditions that predispose affected tissues to malignancy (Sundaram and Prabhu, 2025). In support of this, multiple reports highlight the development of LC in regions of prior granulomas, scarring, or lesions caused by Mtb (Cicėnas and Vencevičius, 2007; Wu et al., 2011).

**Figure 1:**
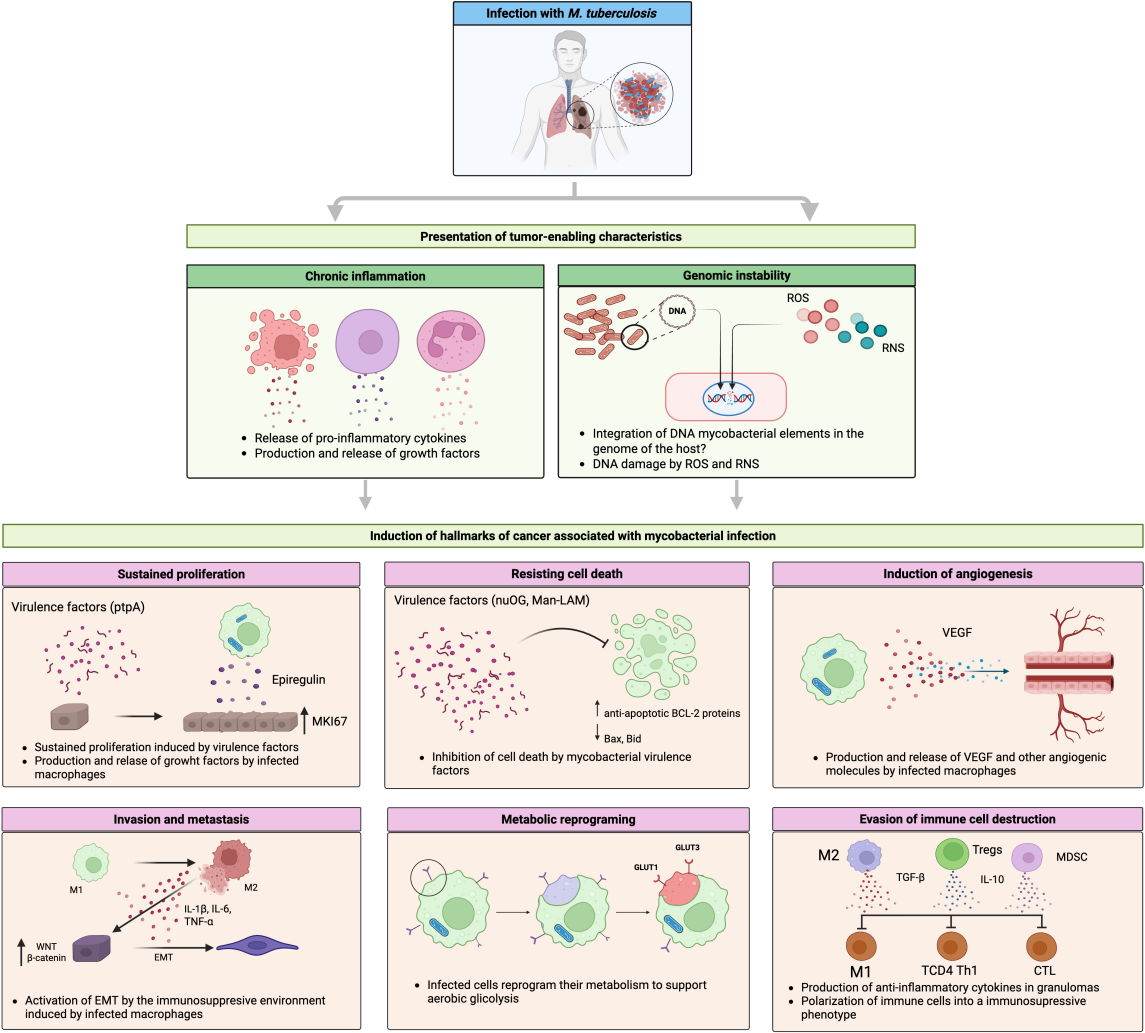
Molecular mechanisms linking *Mycobacterium tuberculosis* infection with lung cancer. *This* infection induces fibrotic lesions that progress to neoplastic. During this process, the presentation of characteristics that favor tumor development, such as chronic inflammation and genomic instability, caused by ROS and RNS released by infected macrophages and other immune cells, creates a microenvironment conducive to the development of incipient tumor cells. Mycobacterial virulence factors (e.g., nuOG or ptpA), along with cytokines, chemokines, and growth factors produced by infected macrophages, contribute to the induction of some features of cancer. This process involves the modulation or dysregulation of key signaling pathways such as MAPK, WNT, NF-kb, and cell death regulated by members of the BCL2 family. Created with BioRender.

**Figure 1** provides a comprehensive depiction of the mechanisms by which *Mycobacterium tuberculosis* (Mtb) infection can promote various cancer hallmarks. Through a sequence of cellular and immunological events, the illustration shows how Mtb fosters chronic inflammation, genomic instability, and remodeling of the pulmonary microenvironment, key elements in malignant transformation. Summarizes how mycobacterial virulence factors and immune mediators activate pathways driving sustained proliferation, apoptosis evasion, angiogenesis, metabolic reprogramming, and immune escape. This establishes a functional link between chronic infection and lung oncogenesis.

Within granulomas, a continuous cycle of cell death and immune cell infiltration has been documented, accompanied by mesenchymal stem cells and a milieu enriched with growth factors and proinflammatory cytokine components that are thought to promote tumorigenic processes (Raghuvanshi et al., 2010).

## Histological and molecular features of tuberculosis-associated lung cancer

A key aspect of this complex interaction is the histological tumor’s subtype. Clinical studies most frequently report cases of pulmonary adenocarcinoma and squamous cell carcinoma in patients with prior TB (Zheng et al., 1987; Abdeahad et al., 2022), suggesting that Mtb induced lesions affect distinct cellular populations and anatomical compartments of the respiratory tract.

Molecularly, specific mutations associated with LC in the context of TB have been identified. A retrospective clinical study in an Asian cohort revealed a strong association between TB-related pulmonary lesions and mutations in the epidermal growth factor receptor (EGFR) gene, specifically exon 19 deletions (Luo et al., 2012). In addition, a study conducted in South Korea reported a significant increase in EGFR exon 19 mutations among patients with LC preceded by TB (Hwang et al., 2019). These findings have important clinical implications, as EGFR-mutant tumors are candidates for treatment based on tyrosine kinase inhibitors (TKIs), offering improved survival outcomes. Current evidence documenting the association between TB and EGFR mutations in LC derives predominantly from Asian populations, where both TB burden and EGFR mutation prevalence are high. Studies from South Korea and Taiwan have reported significant increases in EGFR exon 19 deletions among patients with lung adenocarcinoma preceded by TB. However, comparable studies examining this association in Latin American, African, Middle Eastern, or other non-Asian populations are notably absent from the current literature. This represents a critical knowledge gap, as EGFR mutation frequencies vary substantially across ethnic groups and geographic regions. Future research should prioritize investigation of TB-associated molecular alterations in LC across diverse populations to determine whether the TB-EGFR association is universal or population specific.

However, the presence of TB lesions in patients receiving first generation TKIs correlates with reduced progression-free survival and overall survival. Although the biological mechanisms underlying this phenomenon remain unresolved, it is plausible that chronic inflammation induced by concurrent Mtb infection and cancer may interfere with the therapeutic efficacy of TKIs. Furthermore, production of the epidermal growth factor epiregulin by Mtb infected macrophages may be responsible for squamous metaplasia and tumorigenesis (Nalbandian et al., 2009). Epiregulin has been shown to increase tumor aggressiveness and confer resistance to TKIs (Zhang et al., 2008). These lesions may also promote the emergence of *EGFR* mutations associated with resistance to both first- and second-generation TKIs, a hypothesis that warrants further investigation.

## Biomarkers for predicting lung cancer risk in TB patients

Understanding the molecular mechanisms underlying the TB LC relationship provides a critical foundation for developing biomarkers that identify which Mtb infected individuals face the highest risk for subsequent LC. TB patients have a 2–3 fold increased LC risk compared to controls (SIR 3.20, 95% CI: 2.21-4) (Luczynski et al., 2022), with elevated risk persisting for years after treatment completion, highest in the first year (SIR 4.70) but continuing beyond five years. Since only a subset of TB patients progress to LC, predictive biomarkers would enable targeted surveillance and intervention for high-risk individuals rather than universal screening of all TB survivors, facilitating risk-stratified screening protocols, chemoprevention strategies, and early cancer detection (Feng et al., 2024; Afifah et al., 2025; Fang et al., 2025).

Current research has identified multiple promising biomarker candidates spanning inflammatory, genetic, transcriptomic, metabolic, and epigenetic domains. Integrated multi-biomarker panels show the greatest promise for accurate risk prediction. **Table 3** summarizes the major categories of candidate biomarkers, their specific markers, clinical performance characteristics, and potential applications for LC risk stratification in TB patients. Translation of these discoveries into clinical practice through rigorous validation and implementation research could enable precision prevention strategies, reducing LC burden in TB endemic populations while optimizing resource utilization.

**Table 3:** Independent prognostic factors in coexisting tuberculosis and lung cancer.

Prognostic factor	Impact on outcomes	Key findings
ECOG Performance Status	Negative predictor	ECOG 3-4: HR 12.866 (95% CI: 2.730-60.638) in patients with diagnosis intervals >6 months
Surgical Treatment	Positive predictor	Surgery: HR 0.193 (95% CI: 0.038-0.970); particularly beneficial when diagnosis interval >6 months
Cancer Stage	Negative predictor	Advanced stage (III/IV) significantly worsens prognosis, especially with concurrent TB diagnosis
Age	Negative predictor	Elderly patients (≥65 years) with short diagnosis intervals (<6 months) have particularly poor outcomes
Smoking History	Negative predictor	Smoking compounds mortality risk in TB-LC coexistence
C-Reactive Protein	Biomarker predictor	Elevated CRP (10–50 mg/L or >50 mg/L) indicates worse prognosis

## How tuberculosis affects LC prognosis

The impact of tuberculosis infection on LC prognosis is complex and context-dependent, with outcomes varying based on multiple clinical factors, including the timing of diagnoses, TB disease status (active versus prior infection), histological subtype, treatment approaches, and patient performance status (Liao et al., 2023; Xiong et al., 2023; Zhou et al., 2025).

Patients with a history of treated TB who subsequently develop LC face increased mortality risk. A large cohort study demonstrated that LC patients with prior TB had adjusted hazard ratios of 1.13 (95% CI: 1.04-1.23) for all cause 3 year mortality and 1.11 (95% CI: 1.02-1.21) for cancer specific 3 year mortality compared to LC patients without prior TB (Liao et al., 2023). Importantly, this elevated mortality risk persists regardless of the duration between TB and LC diagnoses; even when more than 3 years separate the two diagnoses, the mortality risk remains elevated.

The timing of diagnosis significantly influences outcomes. Patients diagnosed with both LC and active TB within a ≤6-month interval have a substantially worse prognosis compared to those diagnosed more than 6 months apart. The 1-, 3, and 5 year overall survival rates are 94.2%, 80.3%, and 77.6%, respectively, in the >6 months group versus 88.3%, 63.8%, and 58.5% in the ≤6 months group (HR 0.456, 95% CI: 0.234-0.889) (Xiong et al., 2023).

This difference likely reflects diagnostic delays, advanced disease stage at presentation, and compromised physiological reserve when both diseases are diagnosed simultaneously (Zhou et al., 2025).

Among cancer patients who develop TB, all cause mortality rates are approximately 10.5% at 3 months, 15.56% at 6 months, and 20.56% at 12 months after TB diagnosis, significantly higher than the 11.84% 12 month mortality in cancer patients without TB. Cancer patients with TB had TB direct mortality of 0.83%, higher than the 0.28% in the general population. Respiratory tract cancers show the highest mortality burden (30.15% at 12 months) (Shu et al., 2019).

The prognostic impact of TB varies by LC histology. For squamous cell carcinoma (SCC), the presence of old pulmonary TB lesions is an independent negative prognostic factor, with hazard ratios of 1.72-1.73 (95% CI: 1.12-2.64) for worse survival. The median survival for SCC patients with TB is significantly shorter than those without (1.7 versus 3.4 years) (Zhou et al., 2013).

Conversely, some studies suggest that concomitant active TB in SCC may paradoxically improve survival through enhanced T-cell mediated anti-tumor immunity, with increased CD3+ T cells, CXCR3+ cells and decreased regulatory T cells in the tumor microenvironment (Kuo et al., 2012). In adenocarcinoma, the relationship is less clear and may be modified by EGFR mutation status (Zhou et al., 2013).

Several interconnected biological and clinical mechanisms explain the worse prognosis observed in patients with coexisting TB and LC. First, TB induces persistent chronic inflammation through the release of pro-inflammatory cytokines (TNF-α, IL-1β, IL-6, IL-12, IFN-γ) that promote tumor development, progression, angiogenesis, and epithelial-mesenchymal transition (EMT) (Cao et al., 2019; Budisan et al., 2021; Tan et al., 2021; Qin et al., 2022; Preda et al., 2023).

This chronic inflammatory microenvironment activates NF-κB and STAT3 pathways, facilitating immune escape, DNA damage, and carcinogenesis (Vashishth et al., 2023). Second, TB causes immunosuppression and dysfunction characterized by T cell exhaustion through PD-1/PD-L1 pathway upregulation, which both suppresses anti-TB immunity and promotes tumor immune evasion and metastasis (Kwon et al., 2025; Li et al., 2025), with Mycobacterium tuberculosis antigens directly repressing Th1 mediated anti-tumor immune responses.

Structural lung damage further compounds poor outcomes. Pulmonary TB causes fibrosis, scarring, bronchiectasis, and architectural distortion that create a procarcinogenic microenvironment. This structural damage provides anatomical limitations for cancer treatment, particularly surgical resection, and reduces pulmonary reserve. Additionally, overlapping clinical and radiographic features between TB and LC lead to diagnostic confusion, misdiagnosis, and delayed cancer staging (Lee et al., 2022) resulting in more advanced cancer stage at diagnosis and missed therapeutic windows.

Finally, concurrent anti-TB and anticancer therapy increase toxicity burden, particularly hepatotoxicity (61.3% liver injury in dual therapy patients), hematologic toxicity (grade 3 leukopenia in 9.7%), and compromised performance status (Chai and Shi, 2020; Ye et al., 2020). Drug-drug interactions between rifampicin and targeted cancer therapies further reduce the efficacy of molecularly targeted treatments. Several independent prognostic factors have been identified in patients with coexisting TB and LC (**Table 4**).

**Table 4:** Candidate biomarkers for lung cancer risk prediction in tuberculosis patients.

Biomarker category	Specific markers	Clinical performance / key findings	Potential application
Inflammatory & immune biomarkers			
Circulating Cytokines	IL-6, TNF-α, IL-1β, IL-8	Persistently elevated in TB survivors; correlate with LC stage and prognosis	Identify chronic inflammatory states promoting carcinogenesis
Acute Phase Reactants	C-reactive protein (CRP)	CRP >10 mg/L indicates sustained inflammation; levels 49× normal in post-TB patients	Stratify patients with chronic inflammatory burden
Inflammation-Nutrition Composite	Neutrophil-to-lymphocyte ratio (NLR)	NLR >8 identifies hyperinflammatory state; poor prognosis in TB-LC comorbidity	Risk stratification for surveillance intensity
Inflammation-Nutrition Composite	Prognostic nutritional index (PNI)	PNI 38-50 optimal; lower values predict increased cancer risk	Identify immunocompromised/malnourished high-risk patients
Inflammasome Markers	NLRC4, NLRP3 (plasma)	Combined AUC 0.769 for differentiating TB from TB+NSCLC; higher in TB-only	Early-stage NSCLC/adenocarcinoma detection in TB patients
Transcriptomic & gene expression signatures			
TB-Cancer Transition Genes	KRT80 (Keratin 80)	Upregulated in TB and LUAD; associates with EMT, metastasis, Wnt pathway activation	Monitor molecular transition from TB inflammation to neoplasia
TB-Cancer Transition Genes	C1QTNF6, TRPA1	Opposite expression in TB vs LUAD; prognostic in LUAD	Transition biomarkers for TB-to-cancer progression
Proliferation Marker	MKI67 (Ki-67)	Overexpressed in TB and LUAD; mediates MTB-induced proliferation/invasion	Assess proliferative potential during TB-cancer transition
TB-Related Risk Scores	CD52, CD79A, C1QTNF6, KRT80, GRIA1, TRPA1 signature	AUC 0.68-0.73 across cohorts; predicts survival, immunotherapy response, TME characteristics	Prognostic stratification; treatment selection guidance
Blood-Based mRNA	NPC2 (Niemann-Pick C2)	Distinguishes active TB from LTBI (AUC 0.94-0.95) and TB from LC (AUC 0.86); progressive increase in LTBI→TB transition	Dual-purpose: TB diagnosis and LC differentiation; longitudinal monitoring
Genetic polymorphisms & susceptibility markers			
Mitochondrial Gene Variant	rs4240897 (MFN2)	Inversely associates with TB and LUAD risk (OR 1.31, 95% CI 1.03-1.66)	Identify genetically susceptible individuals for intensive surveillance
MHC Class II Gene	HLA-DQA1 variants	Regulate adaptive immunity; shared TB-LUAD pathways	Genetic risk stratification
Shared Architecture Genes	FHAD1, ZFPM2, DLG2	Represent shared genetic architecture between TB and LUAD	Pathway-based risk assessment
Immune Gene Variants	Interferons, interleukins, TNF, chemokine genes (GWAS)	Predict TB morbidity; may influence cancer progression	Immunogenetic profiling for dual-disease risk
Metabolic & metabolomic biomarkers			
Blood Metabolites (Pro-Carcinogenic)	Oleate, arachidonate, 1-arachidonoylglycerophosphocholine	Associated with increased LC risk in Mendelian randomization studies	Metabolic profiling for carcinogenic risk
Blood Metabolites (Protective)	1-linoleoylglycerophosphoethanolamine, isovalerylcarnitine	Associated with decreased LC risk	Identify protective metabolic states
Urinary Metabolite Profiles	Machine learning-identified biosignatures	Distinguish TB from NTM; predict LC risk with high accuracy	Non-invasive screening tool
AA Metabolism Pathway Genes	PLA2G6, PLA2G7, AKR1C1, AKR1C3, LTA4H, PTGER4	Differentiate sarcoidosis/TB/LC with AUC ≥0.872	Pathway-based disease discrimination
Circulating microRNA biomarkers			
Plasma miRNAs	miR-34a, miR-182	Reduced in both TB and LC vs controls; miR-182 highly significant (p<0.0001)	Diagnosis, prognosis, TB-to-cancer transition monitoring
Multi-miRNA Panels	Combined plasma miRNA signatures	Improve diagnostic accuracy for LC vs TB when bacterial shedding negative	Differential diagnosis in ambiguous clinical presentations
Integrated multi-biomarker models			
Inflammation-Nutrition Model	Diabetes, ECOG, NLR, PNI, HRR, RDW combined	C-index 0.71; 3-year survival AUC 0.693 in TB-LC comorbidity	Comprehensive prognostic assessment
TB Signature Risk Score	Multi-gene TB-LUAD shared DEGs	Validated prognostic value across cohorts; predicts immunotherapy response	Treatment selection; surveillance stratification
Proteomics Risk Model	302-protein prediagnostic panel	AUC 0.75 (vs PLCOm2012 AUC 0.64); 49% sensitivity at 86% specificity	Superior to clinical models and commercial tests

## Safety and feasibility of concurrent therapy

Multiple studies demonstrate that concurrent anti-TB and anti-cancer treatments can be safely and effectively administered (Ye et al., 2020; Wang et al., 2025).

Anti-TB treatment success rates reach 80-87% in LC patients, and cancer treatment completion rates (87.1-92.2%) and response rates (77.4-88.2%) are comparable to LC patients without TB (Chai and Shi, 2020; Ye et al., 2020).

Current evidence suggests initiating anti-TB treatment for 2–4 weeks before commencing immunotherapy or chemotherapy in patients with active TB (Wang et al., 2024; Sun et al., 2025; Wang et al., 2025). For patients with diagnosis intervals >6 months, comprehensive cancer surgery combined with anti-TB therapy yields superior outcomes.

Management recommendations include:

1. High clinical suspicion: LC patients in TB-endemic regions warrant systematic TB screening, particularly those with risk factors (smoking, COPD, immunosuppression).
2. Prompt dual diagnosis: Earlier simultaneous diagnosis and treatment initiation (within appropriate sequencing) improves prognosis compared to delayed recognition.
3. Multidisciplinary approach: Management requires coordinated oncology, pulmonology, infectious disease, and clinical pharmacy collaboration to optimize drug interactions and minimize toxicity.
4. Performance status preservation: Treatment strategies should minimize ECOG deterioration, as this independently predicts mortality.
5. Immunotherapy considerations: Immune checkpoint inhibitors (ICIs) may reactivate latent TB; screening and prophylaxis should precede ICI initiation in high-risk populations.
6. Therapeutic drug monitoring: Essential for managing drug-drug interactions, particularly between rifampicin and tyrosine kinase inhibitors or other molecularly targeted agents.

While TB clearly increases LC risk and affects prognosis through multiple biological mechanisms (chronic inflammation, immune dysregulation, structural damage, and DNA damage), the precise causal pathways and optimal clinical management strategies require further investigation. Future research should focus on elucidating molecular mechanisms linking chronic TB inflammation to specific oncogenic pathways, defining optimal treatment sequencing and timing for concurrent TB cancer therapy, identifying biomarkers predicting which TB patients are at highest LC risk, evaluating cost-effectiveness of latent TB screening and prophylaxis in LC patients, and conducting prospective trials of immune checkpoint inhibitors in TB cancer coexistence.

The bidirectional relationship between these diseases TB promoting carcinogenesis and cancer predisposing to TB underscores the importance of integrated clinical management approaches that address both conditions simultaneously.

## Immune checkpoints and tuberculosis in the context of lung cancer

Immunotherapy has gained increasing relevance in the field of LC due to the presence of immune regulatory molecules, known as checkpoints, within the tumor microenvironment. These checkpoints have become important therapeutic targets. Numerous clinical studies show that immunotherapy in tumors expressing checkpoint molecules is associated with improved survival outcomes. The benefit depends on both the extent and type of immune cell infiltration present.

Recently, it has been reported that patients with active tuberculosis exhibit elevated expression of PD-1 and PD-L1 in CD4^+^ T lymphocytes and monocytes, respectively (Shen et al., 2016a; Shen et al., 2016b). Additionally, several studies have documented increased expression of other checkpoint molecules in active TB, including BTLA, CTLA-4, and TIM-3 (Qiu et al., 2012; Saharia et al., 2016; Zhang et al., 2020).

These findings suggest that Mtb infection may alter the local tumor microenvironment. Such changes could help tumors establish themselves by suppressing the immune response required for their elimination of Mtb. However, whether Mtb infection directly induces checkpoint expression or contributes causally to cancer development remains unresolved.

The use of checkpoint inhibitors in patients with LC carries distinct implications for TB. On one hand, it has been proposed that therapies targeting PD-1, PD-L1, GITR, TIM-3, and CTLA-4 might benefit patients with coexisting LC and TB (Yang et al., 2023). On the other hand, reports indicate that patients treated with immune checkpoint inhibitors are at increased risk of TB or reactivation of (Zaemes and Kim, 2020; Chen et al., 2024).

Therefore, additional studies are urgently needed to deepen our knowledge of the relationship between Mtb infection, immune checkpoint expression, and tumor progression. Clarifying this interplay could enhance both oncologic and infectious disease management strategies.

## Discussion

This work highlights and examines key findings derived from *in vitro* models and patient-based studies that link Mtb infection to an increased risk of developing LC. Current evidence suggests that infection with this pathogen induces cellular changes that lead to genomic instability, sustained proliferative signaling, immune evasion, metabolic reprogramming, angiogenesis, and metastatic potential. The inflammation elicited by the mycobacterium appears to drive both cellular and microenvironmental transformations that favor tumor initiation and establishment. In advanced infection, Mtb induces an immunosuppressive state. This is characterized by polarization of T cells to Th2 and macrophages to M2 phenotypes, both of which may contribute to tumor progression.

Nevertheless, several questions remain unanswered within this field. For instance, the influence and specific association of different Mtb strains on inflammation and subsequent tumorigenesis are not yet fully understood. High-throughput sequencing now allows detailed genotyping and classification of clinical strains from diverse regions. Genetic variations impact metabolism, methylation, cell wall composition, virulence, prognosis, and survival (Borrell et al., 2019). Understanding these associations could allow correlation of strain genotype with virulence and cancer risk, thereby enabling early prognostic assessment and timely interventions to prevent tumor development.

An important aspect to emphasize is the role of Mtb infection in acquiring specific cancer hallmarks. However, comprehensive information is still lacking regarding processes such as epigenetic regulation, phenotypic plasticity, replicative immortality, and microbiome disruptions. Investigating these components could lead to deeper insights into infection-mediated oncogenesis and the identification of potential molecular targets for cancer prevention. Expanding research in this area may offer a clearer understanding of Mtb participation across the stages of carcinogenesis and strengthen the case for its reclassification as a carcinogenic agent.

Clinical evidence supports the hypothesis that Mtb could be considered a potential carcinogen. Pathological studies show a coincidence between tuberculosis-induced inflammatory damage and tumor sites. However, the initial molecular events that trigger the malignant transformation remain poorly defined. Recent transcriptomic research has identified genes with altered expression associated with both tuberculosis and cancer prognosis, which opens the door to the development of clinical biomarkers and early detection through their integration with imaging techniques (Feng et al., 2024).

However, this review recognizes significant limitations. First, most clinical studies are observational and retrospective, which introduces selection biases, confounding, and limitations in establishing causality. Second, there is great heterogeneity between studies in terms of populations, geographical regions, diagnostic criteria, and duration of follow-up. Most of the data comes from East Asia, which restricts generalization to other populations with different genetic and environmental contexts. Third, much of the mechanistic evidence comes from *in vitro* models with transformed cell lines (such as A549) and from animal models, which do not faithfully reproduce the complexity of human lung tissue in chronic infection. Fourth, although multiple candidate biomarkers have been identified, most lack validation in large, independent prospective cohorts, and their clinical utility and cost-effectiveness are not yet demonstrated. Fifth, the temporal relationship between tuberculosis and LC is not fully characterized: the latency period, the dose-response relationship with the severity of the infection, and the impact of treatment on subsequent risk are unknown. Finally, shared confounders such as smoking, socioeconomic status, poor nutrition, environmental exposures, and respiratory comorbidities have not been comprehensively addressed, making it difficult to isolate the independent effect of tuberculosis.

Based on these limitations, research priorities are proposed. Multicenter, prospective, large-scale cohort studies that follow TB patients longitudinally, with standardized clinical, radiological, and biomarker assessments, are required. These studies should include diverse populations and comprehensively collect confounding variables. The creation of biorepositories with serial samples of blood, sputum, and tissue would facilitate the discovery and validation of biomarkers.

In the mechanistic field, it is recommended to extend research to primary bronchial epithelial cells and three dimensional models of organoids that better reproduce the lung microenvironment. The effects of diverse clinical strains, differentiating between latent and active infection, and considering how host genetic polymorphisms modulate susceptibility to carcinogenesis should also be evaluated. Little explored aspects, such as epigenetic modifications, phenotypic plasticity, replicative immortality, and alterations of the microbiome, require priority attention.

In terms of biomarkers, it is necessary to move from the discovery phase to rigorous validations in independent cohorts, with standardized methodologies and sufficient statistical power. Cost-effectiveness analyses comparing biomarker-based strategies versus conventional methods are essential, especially in contexts of high TB burden and limited resources. The development of minimally invasive or point-of-care tests represents a key translational goal.

Intervention studies should explore whether specific TB treatments, anti-inflammatory therapies, or chemoprevention strategies reduce the incidence of LC. Randomized clinical trials evaluating the impact of latent tuberculosis treatment, prolonged anti-inflammatory therapy, or targeted surveillance protocols would provide critical evidence for prevention.

It also proposes to establish harmonized international clinical registries that integrate data from patients with coexisting tuberculosis and LC. These registries would allow analyses with sufficient statistical power to evaluate prognostic factors, therapeutic sequences, drug interactions, and survival outcomes, in addition to incorporating molecular characterization of strains and tumors to advance towards precision medicine.

Health systems research must identify barriers and enablers to implementing integrated screening and management programs, especially in low- and middle-income countries. Comparative economic evaluations of screening algorithms and resource allocation strategies are critical to designing evidence-based policies. Finally, the need for advocacy efforts before international funding and regulatory agencies is underlined to ensure dedicated resources and consider the reclassification of Mtb as a carcinogen by organizations such as the International Agency for Research on Cancer.

## Conclusion

The available evidence consistently supports the association between Mtb infection and an increased risk of LC, particularly adenocarcinoma. Mechanistic studies show that Mtb favors processes of chronic inflammation, genomic instability, angiogenesis, metabolic reprogramming, and immune evasion, all of which are considered hallmarks of carcinogenesis. At the clinical and epidemiological level, patients with previous or concurrent tuberculosis have a higher incidence of LC, a worse prognosis, and specific molecular alterations, such as EGFR mutations. However, gaps in knowledge persist around causality, strain dependent effects, epigenetic regulation, and the differences between latent and active infection. Overcoming these limitations will require multicenter prospective cohorts, advanced experimental models, and rigorous biomarker validation. Recognizing Mtb not only as an infectious pathogen but also as a potential oncogenic agent could transform prevention, early diagnosis, and comprehensive management strategies in endemic regions.

## References

[B1] Abbasnia S. Hashem Asnaashari A. M. Sharebiani H. Soleimanpour S. Mosavat A. Rezaee S. A. (2024). Mycobacterium tuberculosis and host interactions in the manifestation of tuberculosis. J. Clin. Tuberc Mycobact Dis. 36, 100458. doi: 10.1016/j.jctube.2024.100458, PMID: 38983441 PMC11231606

[B2] Abdeahad H. Salehi M. Yaghoubi A. Aalami A. H. Aalami F. Soleimanpour S. (2022). Previous pulmonary tuberculosis enhances the risk of lung cancer: systematic reviews and meta-analysis. Infect. Dis. 54, 255–268. doi: 10.1080/23744235.2021.2006772, PMID: 34807803

[B3] Afifah N. N. Intania R. Wijaya I. Obinata H. Barliana M. I. (2025). The interplay between TB and lung cancer: risk, prognosis and treatment dynamics. Int. J. Tuberc Lung Dis. 29, 243–252. doi: 10.5588/ijtld.24.0491, PMID: 40495306

[B4] Ai J.-W. Ruan Q.-L. Liu Q.-H. Zhang W.-H. (2016). Updates on the risk factors for latent tuberculosis reactivation and their managements. Emerg. Microbes Infect. 5, 1–8. doi: 10.1038/emi.2016.10, PMID: 26839146 PMC4777925

[B5] Ameya G. Birri D. J. (2023). The molecular mechanisms of virus-induced human cancers. Microb. Pathog. 183, 106292. doi: 10.1016/j.micpath.2023.106292, PMID: 37557930

[B6] Ansari T. Brimer N. Vande Pol S. B. (2012). Peptide interactions stabilize and restructure human papillomavirus type 16 E6 to interact with p53. J. Virol. 86, 11386–11391. doi: 10.1128/jvi.01236-12, PMID: 22896608 PMC3457172

[B7] (2024). Global tuberculosis report 2024. 1st ed (Geneva: World Health Organization), 1.

[B8] (2018). Virulence factors and pathogenicity of mycobacterium. Mycobacterium - Res. Dev. doi: 10.5772/intechopen.72027

[B9] Echeverria-Valencia G. Flores-Villalva S. Espitia C. I . List of classifications.

[B10] Bhatt M. Kant S. Bhaskar R. (2012). Pulmonary tuberculosis as differential diagnosis of lung cancer. South Asian J. Cancer 1, 36–42. doi: 10.4103/2278-330X.96507, PMID: 24455507 PMC3876596

[B11] Borrell S. Trauner A. Brites D. Rigouts L. Loiseau C. Coscolla M. . (2019). Reference set of Mycobacterium tuberculosis clinical strains: A tool for research and product development. PloS One 14, e0214088. doi: 10.1371/journal.pone.0214088, PMID: 30908506 PMC6433267

[B12] Bray F. Laversanne M. Sung H. Ferlay J. Siegel R. L. Soerjomataram I. . (2024). Global cancer statistics 2022: GLOBOCAN estimates of incidence and mortality worldwide for 36 cancers in 185 countries. CA Cancer J. Clin. 74, 229–263. doi: 10.3322/caac.21834, PMID: 38572751

[B13] Brewer W. J. Xet-Mull A. M. Yu A. Sweeney M. I. Walton E. M. Tobin D. M. (2022). Macrophage NFATC2 mediates angiogenic signaling during mycobacterial infection. Cell Rep. 41, 111817. doi: 10.1016/j.celrep.2022.111817, PMID: 36516756 PMC9880963

[B14] Budisan L. Zanoaga O. Braicu C. Pirlog R. Covaliu B. Esanu V. . (2021). Links between infections, lung cancer, and the immune system. Int. J. Mol. Sci. 22, 9394. doi: 10.3390/ijms22179394, PMID: 34502312 PMC8431665

[B15] Cao S. Li J. Lu J. Zhong R. Zhong H. (2019). Mycobacterium tuberculosis antigens repress Th1 immune response suppression and promotes lung cancer metastasis through PD-1/PDl-1 signaling pathway. Cell Death Dis. 10. doi: 10.1038/s41419-018-1237-y, PMID: 30718463 PMC6362089

[B16] Carabalí-Isajar M. L. Rodríguez-Bejarano O. H. Amado T. Patarroyo M. A. Izquierdo M. A. Lutz J. R. . (2023). Clinical manifestations and immune response to tuberculosis. World J. Microbiol. Biotechnol. 39. doi: 10.1007/s11274-023-03636-x, PMID: 37221438 PMC10205569

[B17] Chai Q. Lu Z. Liu Z. Zhong Y. Zhang F. Qiu C. . (2020). Lung gene expression signatures suggest pathogenic links and molecular markers for pulmonary tuberculosis, adenocarcinoma and sarcoidosis. Commun. Biol. 3. doi: 10.1038/s42003-020-01318-0, PMID: 33097805 PMC7584606

[B18] Chai M. Shi Q. (2020). The effect of anti-cancer and anti-tuberculosis treatments in lung cancer patients with active tuberculosis: a retrospective analysis. BMC Cancer 20, 1121. doi: 10.1186/s12885-020-07622-6, PMID: 33213414 PMC7678174

[B19] Chen H.-W. Kuo Y.-W. Chen C.-Y. Chang C.-H. Wang S.-M. Chien Y.-C. . (2024). Increased tuberculosis reactivation risk in patients receiving immune checkpoint inhibitor-based therapy. Oncologist 29, e498–e506. doi: 10.1093/oncolo/oyad340, PMID: 38227604 PMC10994249

[B20] Cicėnas S. Vencevičius V. (2007). Lung cancer in patients with tuberculosis. World J. Surg. Oncol. 5. doi: 10.1186/1477-7819-5-22, PMID: 17309797 PMC1805441

[B21] Cohen A. Mathiasen V. D. Schön T. Wejse C. (2019). The global prevalence of latent tuberculosis: a systematic review and meta-analysis. Eur. Respir. J. 54, 1900655. doi: 10.1183/13993003.00655-2019, PMID: 31221810

[B22] Cronan M. R. (2022). In the thick of it: formation of the tuberculous granuloma and its effects on host and therapeutic responses. Front. Immunol. 13. doi: 10.3389/fimmu.2022.820134, PMID: 35320930 PMC8934850

[B23] Da Silva A. L. G. Bresciani M. J. Karnopp T. E. Weber A. F. Ellwanger J. H. Henriques J. A. P. . (2015). DNA damage and cellular abnormalities in tuberculosis, lung cancer and chronic obstructive pulmonary disease. Multidiscip Respir. Med. 10, 38. doi: 10.1186/s40248-015-0034-z, PMID: 26688728 PMC4684909

[B24] Dutta N. K. Karakousis P. C. (2014). Latent tuberculosis infection: myths, models, and molecular mechanisms. Microbiol. Mol. Biol. Rev. 78, 343–371. doi: 10.1128/mmbr.00010-14, PMID: 25184558 PMC4187682

[B25] Dvorak H. F. (1986). Tumors: Wounds that do not heal. Similarities between tumor stroma generation and wound healing. N Engl. J. Med. 315, 1650–1659. doi: 10.1056/nejm198612253152606, PMID: 3537791

[B26] Ehrt S. Schnappinger D. (2009). Mycobacterial survival strategies in the phagosome: defence against host stresses. Cell Microbiol. 11, 1170–1178. doi: 10.1111/j.1462-5822.2009.01335.x, PMID: 19438516 PMC3170014

[B27] Elagan S. K. Almalki S. J. Alharthi M. R. Mohamed M. S. EL-Badawy M. F. (2021). Role of bacteria in the incidence of common GIT cancers: the dialectical role of integrated bacterial DNA in human carcinogenesis. Infect. Drug Resist. 14, 2003–2014. doi: 10.2147/idr.s309051, PMID: 34103947 PMC8179827

[B28] Espina C. Feliu A. Maza M. Almonte M. Ferreccio C. Finck C. . (2023). Latin America and the Caribbean Code Against Cancer 1st Edition: 17 cancer prevention recommendations to the public and to policy-makers (World Code Against Cancer Framework). Cancer Epidemiol. 86, 102402. doi: 10.1016/j.canep.2023.102402, PMID: 37852725

[B29] Fang C. He X. Tang F. Wang Z. Pan C. Zhang Q. . (2025). Where lung cancer and tuberculosis intersect: recent advances. Front. Immunol. 16. doi: 10.3389/fimmu.2025.1561719, PMID: 40242762 PMC11999974

[B30] Feng F. Xu W. Lian C. Wang L. Wang Z. Chen H. . (2024). Tuberculosis to lung cancer: application of tuberculosis signatures in identification of lung adenocarcinoma subtypes and marker screening. J. Cancer 15, 5329–5350. doi: 10.7150/jca.97898, PMID: 39247607 PMC11375533

[B31] Finlay B. B. McFadden G. (2006). Anti-immunology: evasion of the host immune system by bacterial and viral pathogens. Cell 124, 767–782. doi: 10.1016/j.cell.2006.01.034, PMID: 16497587

[B32] Ghosh C. Sarkar A. Anuja K. Das M. C. Chakraborty A. Jawed J. J. . (2019). Free radical stress induces DNA damage response in RAW264.7 macrophages during Mycobacterium smegmatis infection. Arch. Microbiol. 201, 487–498. doi: 10.1007/s00203-018-1587-y, PMID: 30386884

[B33] Girmay G. Kiflie A. Alem M. Lemma M. Bewket G. (2024). Human-immunodeficiency virus infection associated with the impaired Th1 and pro-inflammatory cytokine response in latent tuberculosis-infected individuals: A comparative cross-sectional study. PloS One 19, e0313306. doi: 10.1371/journal.pone.0313306, PMID: 39514524 PMC11548834

[B34] Gupta P. K. Tripathi D. Kulkarni S. Rajan M. G. R. (2016). Mycobacterium tuberculosis H37Rv infected THP-1 cells induce epithelial mesenchymal transition (EMT) in lung adenocarcinoma epithelial cell line (A549). Cell Immunol. 300, 33–40. doi: 10.1016/j.cellimm.2015.11.007, PMID: 26677761

[B35] Hadifar S. Mostafaei S. Behrouzi A. Fateh A. Riahi P. Siadat S. D. . (2021). Strain-specific behavior of Mycobacterium tuberculosis in A549 lung cancer cell line. BMC Bioinf. 22. doi: 10.1186/s12859-021-04100-z, PMID: 33765916 PMC7992940

[B36] Hanahan D. (2022). Hallmarks of cancer: new dimensions. Cancer Discov. 12, 31–46. doi: 10.1158/2159-8290.cd-21-1059, PMID: 35022204

[B37] Hanahan D. Weinberg R. A. (2011). Hallmarks of cancer: The next generation. Cell 144, 646–674. doi: 10.1016/j.cell.2011.02.013, PMID: 21376230

[B38] Hibino S. Kawazoe T. Kasahara H. Itoh S. Ishimoto T. Sakata-Yanagimoto M. . (2021). Inflammation-induced tumorigenesis and metastasis. Int. J. Mol. Sci. 22, 5421. doi: 10.3390/ijms22115421, PMID: 34063828 PMC8196678

[B39] Holla S. Ghorpade D. S. Singh V. Bansal K. Balaji K. N. (2014). Mycobacterium bovis BCG promotes tumor cell survival from tumor necrosis factor-α-induced apoptosis. Mol. Cancer 13. doi: 10.1186/1476-4598-13-210, PMID: 25208737 PMC4174669

[B40] Huang Z.-H. Zhu Y.-F. Zeng Y.-Y. Huang H.-Y. Liu J.-Q. Cen W.-C. . (2025). Pulmonary sarcomatoid carcinoma coexisting with tuberculosis: a case report and literature review. Front. Oncol. 14. doi: 10.3389/fonc.2024.1492574, PMID: 39868375 PMC11757094

[B41] Hunter R. L. Olsen M. Jagannath C. Actor J. K. (2006). Trehalose 6,6′-dimycolate and lipid in the pathogenesis of caseating granulomas of tuberculosis in mice. Am. J. Pathol. 168, 1249–1261. doi: 10.2353/ajpath.2006.050848, PMID: 16565499 PMC1606544

[B42] Hwang I. K. Paik S. S. Lee S. H. (2019). Impact of pulmonary tuberculosis on the EGFR mutational status and clinical outcome in patients with lung adenocarcinoma. Cancer Res. Treat 51, 158–168. doi: 10.4143/crt.2018.084, PMID: 29621876 PMC6333978

[B43] Kumar D. Narayanan S. (2012). pknE, a serine/threonine kinase of Mycobacterium tuberculosis modulates multiple apoptotic paradigms. Infect. Genet. Evol. 12, 737–747. doi: 10.1016/j.meegid.2011.09.008, PMID: 21945589

[B44] Kuo C.-H. Lo C.-Y. Chung F.-T. Lee K.-Y. Lin S.-M. Wang C.-H. . (2012). Concomitant active tuberculosis prolongs survival in non-small cell lung cancer: A study in a tuberculosis-endemic country. PloS One 7, e33226. doi: 10.1371/journal.pone.0033226, PMID: 22438899 PMC3306389

[B45] Kwon K. W. Kang T. G. Lee J. B. Choi E. Kim H. Park M. C. . (2025). Mycobacterium tuberculosis-specific T cells restrain anti-cancer drug-induced neutrophilic lung inflammation in tuberculosis. Nat. Commun. 16, 8875. doi: 10.1038/s41467-025-63930-0, PMID: 41053048 PMC12501240

[B46] Lee H. Y. Kang H. S. Kang J. Y. Kim J. W. Lee S. H. Kim S. J. . (2022). Clinical characteristics and survival of patients concurrently diagnosed with lung cancer and active pulmonary tuberculosis. Transl. Cancer Res. 11, 2671–2680. doi: 10.21037/tcr-22-272, PMID: 36093537 PMC9459664

[B47] Li S. Feng M. Wang F. Liu D. Li M. Dai J. . (2025). Mycobacterium tuberculosis infection may increase the degrees of Malignancy in lung adenocarcinoma. Front. Immunol. 16. doi: 10.3389/fimmu.2025.1537520, PMID: 40061944 PMC11885956

[B48] Li D. Wu M. (2021). Pattern recognition receptors in health and diseases. Signal Transduct Target Ther. 6. doi: 10.1038/s41392-021-00687-0, PMID: 34344870 PMC8333067

[B49] Liao K.-M. Lee C.-S. Wu Y.-C. Shu C.-C. Ho C.-H. (2023). Prior treated tuberculosis and mortality risk in lung cancer. Front. Med. 10. doi: 10.3389/fmed.2023.1121257, PMID: 37064038 PMC10090669

[B50] Liu X. Yin L. Shen S. Hou Y. (2023). Inflammation and cancer: paradoxical roles in tumorigenesis and implications in immunotherapies. Genes Dis. 10, 151–164. doi: 10.1016/j.gendis.2021.09.006, PMID: 37013041 PMC10066281

[B51] Luczynski P. Poulin P. Romanowski K. Johnston J. C. (2022). Tuberculosis and risk of cancer: A systematic review and meta-analysis. PloS One 17, e0278661. doi: 10.1371/journal.pone.0278661, PMID: 36584036 PMC9803143

[B52] Luo Y.-H. Wu C.-H. Wu W.-S. Huang C.-Y. Su W.-J. Tsai C.-M. . (2012). Association between tumor epidermal growth factor receptor mutation and pulmonary tuberculosis in patients with adenocarcinoma of the lungs. J. Thorac. Oncol. 7, 299–305. doi: 10.1097/jto.0b013e31823c588d, PMID: 22173705

[B53] Magcwebeba T. Dorhoi A. Du Plessis N. (2019). The emerging role of myeloid-derived suppressor cells in tuberculosis. Front. Immunol. 10. doi: 10.3389/fimmu.2019.00917, PMID: 31114578 PMC6502992

[B54] Maison D. P. (2022). Tuberculosis pathophysiology and anti-VEGF intervention. J. Clin. Tuberc Mycobact Dis. 27, 100300. doi: 10.1016/j.jctube.2022.100300, PMID: 35111979 PMC8790470

[B55] Malik A. A. Sheikh J. A. Ehtesham N. Z. Hira S. Hasnain S. E. (2022). Can Mycobacterium tuberculosis infection lead to cancer? Call for a paradigm shift in understanding TB and cancer. Int. J. Med. Microbiol. 312, 151558. doi: 10.1016/j.ijmm.2022.151558, PMID: 35842995

[B56] Michels N. van Aart C. Morisse J. Mullee A. Huybrechts I. (2021). Chronic inflammation towards cancer incidence: A systematic review and meta-analysis of epidemiological studies. Crit. Rev. Oncol. Hematol. 157, 103177. doi: 10.1016/j.critrevonc.2020.103177, PMID: 33264718

[B57] Miller J. L. Velmurugan K. Cowan M. J. Briken V. (2010). The type I NADH dehydrogenase of mycobacterium tuberculosis counters phagosomal NOX2 activity to inhibit TNF-α-mediated host cell apoptosis. PloS Pathog. 6, e1000864. doi: 10.1371/journal.ppat.1000864, PMID: 20421951 PMC2858756

[B58] Mohammadnabi N. Shamseddin J. Emadi M. Bodaghi A. B. Varseh M. Shariati A. . (2024). *Mycobacterium tuberculosis*: the mechanism of pathogenicity, immune responses, and diagnostic challenges. J. Clin. Lab. Anal. 38. doi: 10.1002/jcla.25122, PMID: 39593272 PMC11632860

[B59] Nalbandian A. Yan B.-S. Pichugin A. Bronson R. T. Kramnik I. (2009). Lung carcinogenesis induced by chronic tuberculosis infection: the experimental model and genetic control. Oncogene 28, 1928–1938. doi: 10.1038/onc.2009.32, PMID: 19330024

[B60] Pal R. Talwar S. Pandey M. Nain V. K. Sharma T. Tyagi S. . (2024). Rv0495c regulates redox homeostasis in Mycobacterium tuberculosis. Tuberculosis 145, 102477. doi: 10.1016/j.tube.2024.102477, PMID: 38211498

[B61] Piñeros M. Laversanne M. Barrios E. Cancela M. D. C. De Vries E. Pardo C. . (2022). An updated profile of the cancer burden, patterns and trends in Latin America and the Caribbean. Lancet Reg. Health - Am. 13, 100294. doi: 10.1016/j.lana.2022.100294, PMID: 36189115 PMC9483035

[B62] Plummer M. De Martel C. Vignat J. Ferlay J. Bray F. Franceschi S. (2016). Global burden of cancers attributable to infections in 2012: a synthetic analysis. Lancet Glob Health 4, e609–e616. doi: 10.1016/s2214-109x(16)30143-7, PMID: 27470177

[B63] Polena H. Boudou F. Tilleul S. Dubois-Colas N. Lecointe C. Rakotosamimanana N. . (2016). Mycobacterium tuberculosis exploits the formation of new blood vessels for its dissemination. Sci. Rep. 6. doi: 10.1038/srep33162, PMID: 27616470 PMC5018821

[B64] Prasad S. K. Bhat S. Shashank D. Akshatha C. R. Sindhu R. Rachtanapun P. . (2022). Bacteria-mediated oncogenesis and the underlying molecular intricacies: what we know so far. Front. Oncol. 12. doi: 10.3389/fonc.2022.836004, PMID: 35480118 PMC9036991

[B65] Preda M. Tănase B. C. Zob D. L. Gheorghe A. S. Lungulescu C. V. Dumitrescu E. A. . (2023). The bidirectional relationship between pulmonary tuberculosis and lung cancer. Int. J. Environ. Res. Public Health 20, 1282. doi: 10.3390/ijerph20021282, PMID: 36674038 PMC9859200

[B66] Qin Y. Chen Y. Chen J. Xu K. Xu F. Shi J. (2022). The relationship between previous pulmonary tuberculosis and risk of lung cancer in the future. Infect. Agent Cancer 17, 20. doi: 10.1186/s13027-022-00434-2, PMID: 35525982 PMC9078090

[B67] Qiu Y. Chen J. Liao H. Zhang Y. Wang H. Li S. . (2012). Tim-3-expressing CD4+ and CD8+ T cells in human tuberculosis (TB) exhibit polarized effector memory phenotypes and stronger anti-TB effector functions. PloS Pathog. 8, e1002984. doi: 10.1371/journal.ppat.1002984, PMID: 23144609 PMC3493466

[B68] Queval C. J. Song O.-R. Carralot J.-P. Saliou J.-M. Bongiovanni A. Deloison G. . (2017). Mycobacterium tuberculosis controls phagosomal acidification by targeting CISH-mediated signaling. Cell Rep. 20, 3188–3198. doi: 10.1016/j.celrep.2017.08.101, PMID: 28954234 PMC5637157

[B69] Raghuvanshi S. Sharma P. Singh S. Van Kaer L. Das G. (2010). *Mycobacterium tuberculosis*evades host immunity by recruiting mesenchymal stem cells. Proc. Natl. Acad. Sci. 107, 21653–21658. doi: 10.1073/pnas.1007967107, PMID: 21135221 PMC3003090

[B70] Ramon-Luing L. Palacios Y. Ruiz A. Téllez-Navarrete N. Chavez-Galan L. (2023). Virulence factors of mycobacterium tuberculosis as modulators of cell death mechanisms. Pathogens 12, 839. doi: 10.3390/pathogens12060839, PMID: 37375529 PMC10304248

[B71] Saharia K. K. Petrovas C. Ferrando-Martinez S. Leal M. Luque R. Ive P. . (2016). Tuberculosis therapy modifies the cytokine profile, maturation state, and expression of inhibitory molecules on mycobacterium tuberculosis-specific CD4+ T-cells. PloS One 11, e0158262. doi: 10.1371/journal.pone.0158262, PMID: 27367521 PMC4930205

[B72] Scriba T. J. Maseeme M. Young C. Taylor L. Leslie A. J. (2024). Immunopathology in human tuberculosis. Sci. Immunol. 9. doi: 10.1126/sciimmunol.ado5951, PMID: 39671470

[B73] Shen L. Gao Y. Liu Y. Zhang B. Liu Q. Wu J. . (2016a). PD-1/PD-L pathway inhibits M.tb-specific CD4+ T-cell functions and phagocytosis of macrophages in active tuberculosis. Sci. Rep. 6. doi: 10.1038/srep38362, PMID: 27924827 PMC5141449

[B74] Shen L. Shi H. Gao Y. Ou Q. Liu Q. Liu Y. . (2016b). The characteristic profiles of PD-1 and PD-L1 expressions and dynamic changes during treatment in active tuberculosis. Tuberculosis 101, 146–150. doi: 10.1016/j.tube.2016.10.001, PMID: 27865385

[B75] Shu C.-C. Liao K.-M. Chen Y.-C. Wang J.-J. Ho C.-H. (2019). The burdens of tuberculosis on patients with Malignancy: incidence, mortality and relapse. Sci. Rep. 9, 11901. doi: 10.1038/s41598-019-48395-8, PMID: 31417132 PMC6695428

[B76] Subbian S. Tsenova L. Kim M.-J. Wainwright H. C. Visser A. Bandyopadhyay N. . (2015). Lesion-specific immune response in granulomas of patients with pulmonary tuberculosis: A pilot study. PloS One 10, e0132249. doi: 10.1371/journal.pone.0132249, PMID: 26133981 PMC4489805

[B77] Sun M. Ji H. Liu A. Xu N. (2025). Concurrent active pulmonary tuberculosis and small cell lung cancer: diagnostic challenges and therapeutic insights from a case report. Front. Oncol. 15. doi: 10.3389/fonc.2025.1564686, PMID: 40978039 PMC12444658

[B78] Sundaram K. Prabhu V. (2025). Contagious illness of tuberculosis and correlation with various types of cancer. Med. Microecol 24, 100123. doi: 10.1016/j.medmic.2025.100123

[B79] Tan Z. Xue H. Sun Y. Zhang C. Song Y. Qi Y. (2021). The role of tumor inflammatory microenvironment in lung cancer. Front. Pharmacol. 12. doi: 10.3389/fphar.2021.688625, PMID: 34079469 PMC8166205

[B80] Tanooka H. Inoue A. Takahashi R. Tatsumi K. Fujikawa K. Nagao T. . (2020). Bacterial SOS Genes *mucAB/umuDC* Promote Mouse Tumors by Activating Oncogenes *Nedd9/Aurkb via* a miR-145 Sponge. Mol. Cancer Res. 18, 1271–1277. doi: 10.1158/1541-7786.mcr-20-0137, PMID: 32513897

[B81] Vandeven N. Nghiem P. (2014). Pathogen-driven cancers and emerging immune therapeutic strategies. Cancer Immunol. Res. 2, 9–14. doi: 10.1158/2326-6066.cir-13-0179, PMID: 24778160 PMC4135058

[B82] Vashishth A. Shuaib M. Bansal T. Kumar S. (2023). Mycobacterium tubercular mediated inflammation and lung carcinogenesis: connecting links. OBM Genet. 07, 1–17. doi: 10.21926/obm.genet.2302183

[B83] Volkman H. E. Pozos T. C. Zheng J. Davis J. M. Rawls J. F. Ramakrishnan L. (2010). Tuberculous granuloma induction *via* interaction of a bacterial secreted protein with host epithelium. Science 327, 466–469. doi: 10.1126/science.1179663, PMID: 20007864 PMC3125975

[B84] Wang H. Gao L. Cai X. Li J. Lang Y. Zheng R. . (2025). Simultaneous anti-tuberculosis and anti-tumor treatment with immune checkpoint inhibitors for co-existent pulmonary tuberculosis and advanced lung cancer. Infect. Drug Resist. 18, 107–112. doi: 10.2147/IDR.S497006, PMID: 39803310 PMC11721805

[B85] Wang C. Zou R.-Q. He G.-Z. (2024). Progress in mechanism-based diagnosis and treatment of tuberculosis comorbid with tumor. Front. Immunol. 15. doi: 10.3389/fimmu.2024.1344821, PMID: 38298194 PMC10827852

[B86] Wojtas B. Fijalkowska B. Wlodarczyk A. Schollenberger A. Niemialtowski M. Hamasur B. . (2011). Mannosylated lipoarabinomannan balances apoptosis and inflammatory state in mycobacteria-infected and uninfected bystander macrophages. Microb. Pathog. 51, 9–21. doi: 10.1016/j.micpath.2011.03.004, PMID: 21440050

[B87] Wong J. Y. Y. Zhang H. Hsiung C. A. Shiraishi K. Yu K. Matsuo K. . (2020). Tuberculosis infection and lung adenocarcinoma: Mendelian randomization and pathway analysis of genome-wide association study data from never-smoking Asian women. Genomics 112, 1223–1232. doi: 10.1016/j.ygeno.2019.07.008, PMID: 31306748 PMC6954985

[B88] Wu C. Hu H. Pu C. Huang N. Shen H. Li C. . (2011). Pulmonary tuberculosis increases the risk of lung cancer: A population-based cohort study. Cancer 117, 618–624. doi: 10.1002/cncr.25616, PMID: 20886634

[B89] Xander C. Rajagopalan S. Jacobs W. R. Braunstein M. (2024). The SapM phosphatase can arrest phagosome maturation in an ESX-1 independent manner in *Mycobacterium tuberculosis* and BCG. Infect. Immun. 92. doi: 10.1128/iai.00217-24, PMID: 38884474 PMC11238552

[B90] Xiong K. Sun W. He Y. Fan L. (2021). Advances in molecular mechanisms of interaction between Mycobacterium tuberculosis and lung cancer: a narrative review. Transl. Lung Cancer Res. 10, 4012–4026. doi: 10.21037/tlcr-21-465, PMID: 34858788 PMC8577982

[B91] Xiong M. Xie S. Wang Y. Cai C. Sha W. Cui H. . (2023). The diagnosis interval influences risk factors of mortality in patients with co-existent active tuberculosis and lung cancer: a retrospective study. BMC Pulm Med. 23, 382. doi: 10.1186/s12890-023-02674-3, PMID: 37817103 PMC10563245

[B92] Yang L. Zhuang L. Ye Z. Li L. Guan J. Gong W. (2023). Immunotherapy and biomarkers in patients with lung cancer with tuberculosis: Recent advances and future Directions. iScience 26, 107881. doi: 10.1016/j.isci.2023.107881, PMID: 37841590 PMC10570004

[B93] Ye M.-F. Su S. Huang Z.-H. Zou J.-J. Su D.-H. Chen X.-H. . (2020). Efficacy and safety of concurrent anti-tuberculosis treatment and chemotherapy in lung cancer patients with co-existent tuberculosis. Ann. Transl. Med. 8, 1143–1143. doi: 10.21037/atm-20-5964, PMID: 33240992 PMC7576042

[B94] Yu M.-J. Li P.-J. Liang Z.-A. (2021). Coexistence of lung squamous cell carcinoma and pulmonary tuberculosis within a single lesion: A case report. OncoTargets Ther. 14, 2575–2578. doi: 10.2147/ott.s302996, PMID: 33880036 PMC8053494

[B95] Yu Y.-H. Liao C.-C. Hsu W.-H. Chen H.-J. Liao W.-C. Muo C.-H. . (2011). Increased lung cancer risk among patients with pulmonary tuberculosis: A population cohort study. J. Thorac. Oncol. 6, 32–37. doi: 10.1097/jto.0b013e3181fb4fcc, PMID: 21150470

[B96] Zaemes J. Kim C. (2020). Immune checkpoint inhibitor use and tuberculosis: a systematic review of the literature. Eur. J. Cancer 132, 168–175. doi: 10.1016/j.ejca.2020.03.015, PMID: 32375103

[B97] Zhang J. Iwanaga K. Choi K. C. Wislez M. Raso M. G. Wei W. . (2008). Intratumoral epiregulin is a marker of advanced disease in non–small cell lung cancer patients and confers invasive properties on*EGFR*-mutant cells. Cancer Prev. Res. (Phila Pa) 1, 201–207. doi: 10.1158/1940-6207.capr-08-0014, PMID: 19138957 PMC3375599

[B98] Zhang J.-A. Lu Y.-B. Wang W.-D. Liu G.-B. Chen C. Shen L. . (2020). BTLA-expressing dendritic cells in patients with tuberculosis exhibit reduced production of IL-12/IFN-α and increased production of IL-4 and TGF-β, favoring th2 and foxp3+ Treg polarization. Front. Immunol. 11. doi: 10.3389/fimmu.2020.00518, PMID: 32296431 PMC7136538

[B99] Zhang H. Ouyang H. Wang D. Shi J. Ouyang C. Chen H. . (2015). Mycobacterium tuberculosis Rv2185c contributes to nuclear factor-κB activation. Mol. Immunol. 66, 147–153. doi: 10.1016/j.molimm.2015.02.020, PMID: 25771181

[B100] Zhang F. Qi F. Han Y. Yang H. Wang Y. Wang G. . (2025). Clinical and imaging features of co-existent pulmonary tuberculosis and lung cancer: a population-based matching study in China. BMC Cancer 25, 89. doi: 10.1186/s12885-024-13350-y, PMID: 39815214 PMC11734471

[B101] Zheng W. Blot W. Liao M. Wang Z. Levin L. Zhao J. . (1987). Lung cancer and prior tuberculosis infection in Shanghai. Br. J. Cancer 56, 501–504. doi: 10.1038/bjc.1987.233, PMID: 2825752 PMC2001820

[B102] Zhou Y. Cui Z. Zhou X. Chen C. Jiang S. Hu Z. . (2013). The presence of old pulmonary tuberculosis is an independent prognostic factor for squamous cell lung cancer survival. J. Cardiothorac Surg. 8, 123. doi: 10.1186/1749-8090-8-123, PMID: 23647947 PMC3670994

[B103] Zhou Y. Hu Z. Cao S. Yan B. Qian J. Zhong H. (2017). Concomitant Mycobacterium tuberculosis infection promotes lung tumor growth through enhancing Treg development. Oncol. Rep. 38, 685–692. doi: 10.3892/or.2017.5733, PMID: 28627635 PMC5561997

[B104] Zhou W. Lu H. Lin J. Zhu J. Liang J. Xie Y. . (2025). Coexisting lung cancer and pulmonary tuberculosis: A comprehensive review from incidence to management. Cancer Rep. Hoboken NJ 8, e70213. doi: 10.1002/cnr2.70213, PMID: 40347011 PMC12065023

[B105] Zhou Z.-J. Xie H.-Y. Bertolaccini L. Xia N. Zhang Y. (2025). Research progress on lung cancer complicated with pulmonary tuberculosis: a narrative review. Transl. Lung Cancer Res. 14, 2272–2280. doi: 10.21037/tlcr-2025-450, PMID: 40673073 PMC12261255

